# Psoas Major Muscle Volume Does Not Affect the Postoperative Thigh Symptoms in XLIF Surgery

**DOI:** 10.3390/brainsci11030357

**Published:** 2021-03-11

**Authors:** Wicharn Yingsakmongkol, Waranyoo Wathanavasin, Khanathip Jitpakdee, Weerasak Singhatanadgige, Worawat Limthongkul, Vit Kotheeranurak

**Affiliations:** 1 Department of Orthopaedics, Faculty of Medicine, Chulalongkorn University (Thai Red Cross Society), Bangkok 10330, Thailand; wicharn707@gmail.com (W.Y.); dr.weerasaks@gmail.com (W.S.); dr.worawat@gmail.com (W.L.); 2 Department of Orthopaedics, Somdej Phra Phutthaloetla Hospital, Mae Klong, Samut Songkram 75000, Thailand; wayanyoowaranyoo@gmail.com; 3 Department of Orthopedics, Queen Savang Vadhana Memorial Hospital (Thai Red Cross Society), Sriracha, Chonburi 20110, Thailand; khanathip.j@gmail.com

**Keywords:** extreme lateral interbody fusion, lateral lumbar interbody fusion (LLIF), neurosurgical procedures, postoperative thigh symptoms, psoas major muscle volume, spine

## Abstract

Background: Extreme lateral interbody fusion (XLIF) is a minimally invasive surgery that accesses the lumbar spine through the psoas muscle. This study aimed to evaluate the correlation between the psoas major muscle volume and anterior thigh symptoms after XLIF. Methods: Eighty-one patients (mean age 63 years) with degenerative spine diseases underwent XLIF (total = 94 levels). Thirty-eight patients were female (46.9%), and 24 patients (29.6%) had a history of lumbar surgery. Supplemental pedicle screws were used in 48 patients, and lateral plates were used in 28 patients. Neuromonitoring devices were used in all cases. The patients were classified into two groups (presence of thigh symptoms and no thigh symptoms after the surgery). The psoas major volumes were measured and calculated by CT (computed tomography) scan and compared between the two patient groups. Results: In the first 24 h after surgery, 32 patients (39.5%) had thigh symptoms (20 reported pain, 9 reported numbness, and 18 reported weakness). At one year postoperatively, only 3 of 32 patients (9.4%) had persistent symptoms. Conclusions: As a final observation, no statistically significant difference in the mean psoas major volume was found between the group of patients with new postoperative anterior thigh symptoms and those with no thigh symptoms. Preoperative psoas major muscle volume seems not to correlate with postoperative anterior thigh symptoms after XLIF.

## 1. Introduction

Extreme lateral interbody fusion (XLIF) is used to gain access to the lumbar spine via a lateral approach that passes through the psoas major muscle. This procedure is an emerging technology in minimally invasive surgery that aims to lower the risks and morbidities associated with conventional posterior and anterior approaches to the spine, such as soft tissue injury, large amount of blood loss, surgical wound complication, and prolonged recovery time [1,2,3,4]. XLIF results in a secure and effective interbody fusion and allows an indirect decompression of neural structures, restoring foramen dimensions and disc space height with a clear clinical improvement for patients [1,4]. However, the procedure requires access through the psoas major muscle, where the lumbar plexus is located. When approaching near the nerve plexus, a process of degenerative cellular and molecular changes due to injury of the nerve could occur, either from direct mechanical insult of the surgical approach or insertion of the instrument or from the reduction of blood supply and oxygen [5]. This leads to neuropathic pain, which could manifest as variety of symptoms such as pain associated with positive or negative sensory signs [6]. As a result, XLIF can possibly result in lumbar plexus injury and lead to anterior thigh pain, sensory changes in the upper thigh and groin areas, and weakness in hip flexors. Psoas major muscle volume, retraction time, levels of vertebrae involved in the procedure, and operative time may be the predictive factors of postoperative complications of XLIF. However, there is still no consensus in previous studies regarding these factors [7,8,9].

Postoperative thigh symptoms including pain, sensory disturbances and weakness are frequently reported following XLIF procedure, especially at L4–5 [10,11]. We hypothesized that the larger psoas muscle volume is associated with higher incidence of postoperative thigh symptoms, possibly due to more extensive muscle injury and more retraction force [10]. The purpose of this study was to evaluate the effect of psoas major muscle volume on postoperative thigh symptoms following the XLIF procedure.

## 2. Materials and Methods

Institutional review board approval was obtained for this study. Postoperative clinical data were retrospectively collected from 81 consecutive patients treated with XLIF (NuVasive Inc., San Diego, CA, USA) at our institution between 2013 and 2018. Patients were scheduled for surgeries only after failure of three months of conservative therapy. Complete imaging studies, including dynamic (flexion, extension) conventional radiographs, CT (computed tomography), and magnetic resonance imaging (MRI) were obtained from every patient.

The mean age at the time of surgery was 63 years (range, 42–91 years) and 56% were female. Twenty-four patients had a history of lumbar surgery. All patients were treated for degenerative lumbar conditions, most commonly from adjacent segmental disease (27), degenerative disc disease (14), spondylolisthesis (23), degenerative scoliosis (11), and spinal canal stenosis (4). Age and sex were similar between the two patient groups. Demographic data of patients are shown in Table 1.

**Figure 1:**
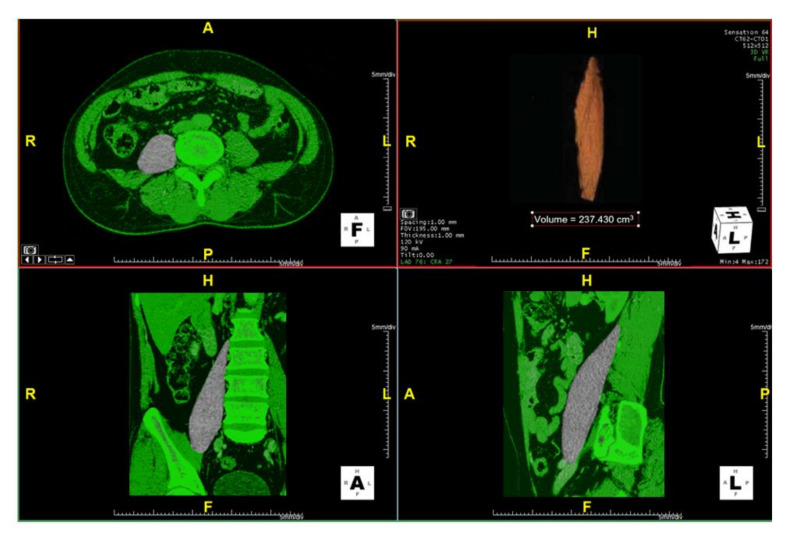
Images demonstrating the method of the psoas major muscle volume measurement by a CT-based software. Measurements are from the origin of the psoas major at the level of the lumbar vertebrae to its insertion in the lesser trochanter on CT datasets using a semi-automatic segmentation method. (A, anterior; P, posterior; R, right; L, left; H, head; F, foot).

A single trained spine fellow (WW) who was blinded to the patients’ clinical data measured the left psoas major muscle volume. The measurement was based on the origin of the psoas major at the level of the lumbar vertebrae to its insertion in the lesser trochanter on CT imaging using a semi-automatic segmentation method (Aquarius iNtuition version 4.4.6, TeraRecon Inc., San Francisco, CA, USA) (Figure 1).

**Figure 2:**
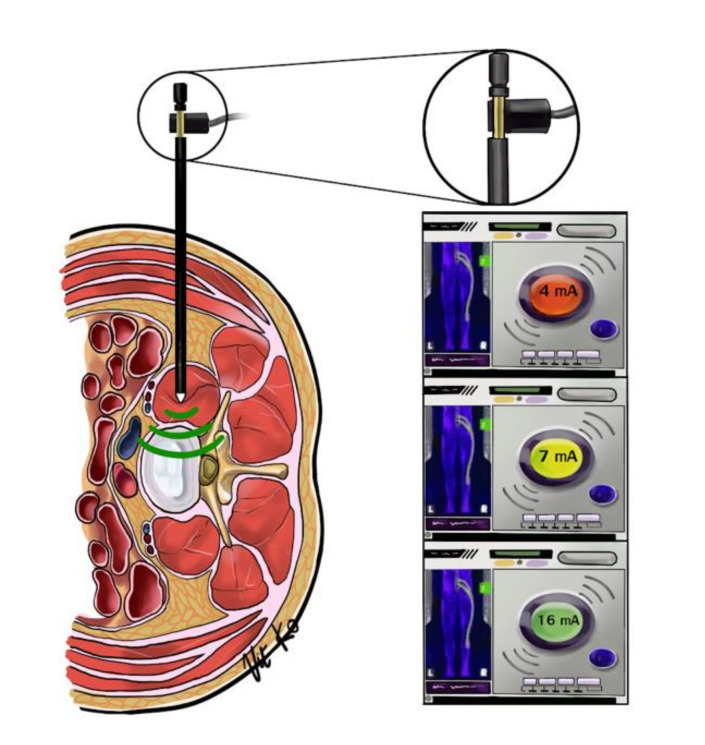
Depicting a neuromonitoring device attached to a serial dilator while gaining a direct access through the psoas major muscle. Note that there are three level of alertness: safe from lumbosacral plexus (green), caution (yellow), and close to the nerve (red).

All XLIF procedures were performed by a single senior surgeon (WY). After general anaesthesia was administered, electrodes were attached for a triggered real-time electromyography (EMG) neuromonitoring (NuVasive, San Diego, CA, USA). The patient was then positioned in the right lateral decubitus position with the iliac crest over the table break, and adhesive tape was used to strap the patient’s body to the operative table. Fluoroscopy was used to identify the level of operation, and the skin was marked before the area was prepped with aseptic solution and draping. A 4-cm skin incision was made at the flank area, blunt dissection was performed to palpate the retroperitoneal fat, and the psoas major muscle was directly identified. Probe and serial dilators attached to the continuous triggered EMG neuromonitoring were used to gain access through the psoas muscle. To avoid lumbar plexus injury, neuro-monitoring (Figure 2) and direct visualization during splitting of the psoas muscle were employed [12]. A guide sensor of the neuro-monitoring device was placed in the approach area. If a green light was shown on the monitor, there was no lumbar plexus in close proximity to the approach area. If the light was yellow, the approach proceeded with caution. If the light was red, the sensor was directly placed on the lumbar plexus. We accepted only the green light on the monitor to proceed with the operation.

After placement and adjustment of the self-retaining retractor allowing direct visualization of the intervertebral disc area, a complete discectomy was performed. The endplate preparation was carried out with caution to minimize the risk of intraoperative endplate injury, which can lead to an early postoperative cage subsidence. Trials were inserted, and a proper sized polyethylethylketone (PEEK) filled with demineralized bone matrix (Attrax, NuVasive Inc., San Diego, CA, USA) was then impacted in a proper position and confirmed by fluoroscopy. The lateral plate was securely fixed at this step (24 patients). The retractor was removed; at this point, the bleeding was thoroughly controlled and the skin was closed in a subcutaneous fashion with no drain retention. The patient was subsequently placed in the prone position, and bilateral percutaneous pedicle-screw fixation was performed under fluoroscopy guidance (Precept, NuVasive Inc., San Diego, CA, USA) (48 patients).

Within 24 h after surgery, all patients were examined and asked whether they developed any new symptoms such as anterior thigh pain, numbness at the groin or front thigh, and weakness of the hip and thigh muscles. The psoas weakness was evaluated using the motor score (Lovett scale) for hip flexor motor strength. Patients were considered having psoas weakness if they had motor strength less than grade 4 (full range of motion against gravity with some resistance). The visual analogue score (VAS), pinprick sensation, and motor grading were used to evaluate these symptoms. The patients were classified into two groups (presence of thigh symptoms group and no thigh symptoms). Both groups were scheduled for regular follow-up visits at 1, 3, 6, and 12 months and the status of the thigh symptoms was recorded.

The psoas major volumes were compared between the two patient groups. Independent t-test and Fisher’s exact test were used for comparisons between the two groups. Baseline demographic and treatment variables were analyzed on a per-patient basis (*n* = 81), whereas analysis of intraoperative variables was performed on a per-patient basis (*n* = 90). Statistical significance was set at *p* < 0.05. Analyses were performed using SPSS v.21 software (IBM SPSS Statistics; IBM Corp, Armonk, NY, USA). All statistical analyses were verified by the institute biostatistician.

## 3. Results

A total of 94 disc-levels were treated (range 1–3 levels per patient). Most of the patients were treated at one level (87.7%). The treated levels were as following; L1–2 (6, 7.4%), L2–3 (21, 25.9%), L3–4 (13, 16.0%), L4–5 (31, 38.3%), L3–5 (7, 8.6%), and L2–5 (3, 3.7%) (Table 2). All intraoperative neuromonitoring showed no abnormal changes of the somatosensory evoked potential (SEP), motor evoked potentials (MEP), and the electromyography (EMG) during the procedures.

Supplemental lateral plate fixation was used in 24 patients (29.6%), pedicle screw and rod fixation was used in 48 patients (59.3%), and XLIF alone was performed in 9 patients (11.1%) (Table 1). None of the patients had an intraoperative estimated blood loss (EBL) of >20 mL.

New postoperative anterior thigh symptoms were found in 32 of 81 patients (39.5%) within 24 h postoperatively; 20 reported pain, 9 reported numbness, and 18 reported weakness (Table 3). Patient-level descriptions of thigh complications and resolution are shown in Table 4. None of the six patients with multiple levels of XLIF experienced anterior thigh symptoms. In the thigh symptom group, the maximum VAS score was 5 out of 10, and the worst motor grade was 4 out of 5. Only 3 of 32 patients (9.4%) had persistent symptoms at 12 months postoperatively: pain in two patients and weakness in one patient.

**Figure 3:**
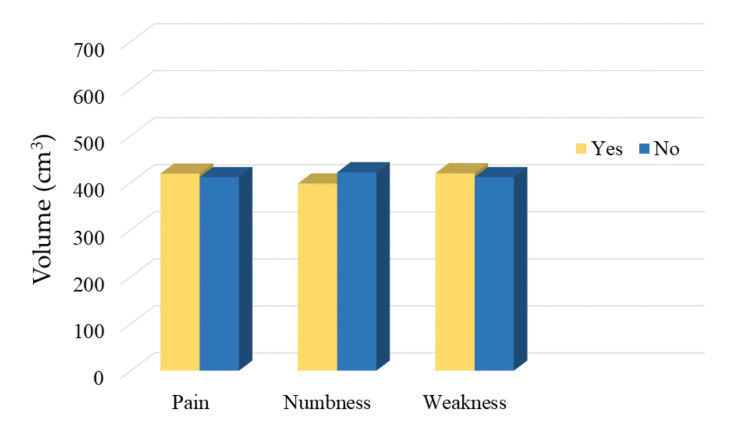
Comparison of psoas major muscle volume of the two groups.

There was no significant difference in mean psoas major muscle volumes between symptomatic patients and asymptomatic patients, as shown in Table 5. In the comparison between the asymptomatic group and each type of symptomatic group, patients with postoperative numbness had a mean psoas major muscle volume smaller than asymptomatic patients. The other patients with postoperative pain and weakness had a mean psoas major muscle volume approximately equal to that of asymptomatic patients (Figure 3).

## 4. Discussion

In previous studies, XLIF resulted in less blood loss, lower complication rates, shorter hospital stays, and faster return to normal activities than the open approach [1,2,3,4]. However, XLIF is a procedure not without risk of complications. Potential adverse events such as lumbar plexus injury leading to postoperative neurological deficits have been reported. Following the surgical approach and application of the surgical instrument, the lumbar plexus could be injured from the direct mechanical insult and reduction of blood and oxygen supply [5]. The nerve injury could lead to neuropathic pain, which is characterized by a variety of positive and negative signs and symptoms such as burning pain, electrical shock-like sensation, tingling, allodynia, or hyperalgesia [13]. Psoas major muscle injury and other soft tissue damages have been responsible for nociceptive and inflammatory pain as a response to harmful stimuli [14]. Thus, one of the most frequent postoperative complications following XLIF was due to the surgical approach through the psoas major muscle during surgery, which can damage the muscle itself or also the lumbar plexus. Injuries to this lumbar plexus following XLIF were mostly partial and healed spontaneously, and the muscle unit territories could regain normal size [15]. These injuries were usually resolved within three months. Although symptoms are usually transient and do not lead to significant morbidity, they can affect clinical outcome, psychological condition, and patient satisfaction. Studies regarding the association between the psoas major muscle and postoperative thigh symptoms and detail of psoas major anatomy are needed to address the risk and lead to reduction of complications [7,16]. Different psoas major characteristics can lead to different degrees of injury to the muscle or nerve following XLIF [17]. Our study verified the hypothesis that larger psoas major muscle volume may be associated with a higher incidence of postoperative thigh symptoms. No associations were found in this regard in the examined group of patients.

Previous literature reported varying rates of postoperative neurologic events (motor or sensory) between 0.6% and 33.6% [3,12,18,19]. In this study, we found a higher rate of postoperative neurologic events (37.7%, not including anterior thigh pain). This might be related to anxiety of postoperative pain causing a falsely low motor grade. In a series of 235 patients with a total of 444 levels, Pumberger et al. [18] reported that the prevalence of sensory deficits was 1.6% at 12 months’ follow-up, which was similar to our study (Table 2). However, in another large series of 600 patients, Rodgers et al. found a lower rate of permanent neurological injury (0.7%) [20].

Regarding psoas major muscle volume and anterior thigh symptoms, Buric et al. studied 29 patients and concluded that patients with postoperative events had significantly less latero-lateral psoas major muscle length measured on axial MRI images than that of patients without postoperative events [16]. To the best of our knowledge, our study is the first to investigate the volume of whole psoas major muscle and its correlation with postoperative thigh symptoms. The method of using imaging software analysis also improved the accuracy of the muscle volume measurement. In contrast to previous studies, this measurement technique represented the whole psoas major muscle volume better than using the diameter in a single plane. However, in the examined group, the results showed that the mean volume of the psoas major muscle was not significantly different between patients with and without postoperative anterior thigh pain, numbness, and weakness. Thus, further studies to investigate the correlation between the psoas major muscle and postoperative anterior thigh symptoms should emphasize other characteristics such as the shape or position of the muscle, location of the intra-psoas neural element, or other factors. As the investigation of the correlation between psoas major muscle factors and postoperative thigh symptoms continues, many prevention strategies, including minimizing retraction time, operative time, appropriate surgical corridor, and use of intraoperative neuromonitoring, have been suggested. Additionally, it is important that all patients should be advised before the operation that these complications can be expected postoperatively but are usually transient.

This study has several limitations. First, the sample size of patients with postoperative thigh symptoms in this study was too small for a clinical correlation to be identified. Thus, our results should be verified on a larger patient group. Second, the enrolled patients underwent different additional procedures (XLIF with percutaneous pedicle screw (PPS), XLIF with XLP|^®^) and different operative levels. Third, these symptoms were subjective, and each patient may have reported the same symptoms differently. Moreover, there may be many significant contributing factors of postoperative thigh symptoms other than psoas major muscle characteristics, as these events were also found in other psoas-preserved approaches, including anterior (ALIF), oblique (OLIF), transforaminal (TLIF), and posterior lumbar interbody fusion (PLIF) [10,21]. Different surgical intervention levels, numbers of fusion levels and status of previous lumbar surgery could be considered as possible confounding factors in the study [8,11]. Patients who had previous lumbar surgery possibly have less muscle volume due to either approach-related muscle injury or periods of inactivity postoperatively. Pressure against the anterior thigh from prone positioning during the surgery could also lead to thigh symptoms [22]. Moreover, degenerative spinal conditions and spinopelvic parameters were reported to affect the lumbar muscle volume, including psoas major as it originates from the transverse processes of T12–L5, and possibly cause functional deficits in the skeletal muscles [23].

## 5. Conclusions

XLIF is a popular minimally invasive technique for achieving spinal fusion in patients with degenerative lumbar spine disease. Preoperative psoas major muscle volume was not found to be correlated with postoperative anterior thigh numbness, pain, or weakness. Additionally, most of these complications resolved without any intervention. Further studies with more subjects should focus on characteristics of the psoas major muscle other than its volume, such as the muscle morphology or fatty infiltration, or identify other risk factors in order to reduce complication rates associated with this procedure.

## Figures and Tables

**Table 1:** Patient demographic information (*n* = 81).

	N (%)
Female	38 (46.9)
History of previous lumbar surgery	24 (29.6)
Diagnosis	
- ASD	27 (33.3)
- Spondylolisthesis	23 (28.4)
- DDD	14 (17.3)
- Degenerative scoliosis	11 (13.6)
- Spinal canal stenosis	4 (4.9)
Operation	
- XLP	24 (29.6)
- PPS	48 (59.3)
- Alone	9 (11.1)

ASD, adjacent segmental disease; DDD, degenerative disc disease; XLP, lateral plate fixation; PPS, percutaneous screw fixation.

**Table 2:** Comparison of mean age and levels of surgery between symptomatic and asymptomatic patients.

	Symptomatic Patients (*n* = 30)	Asymptomatic Patients (*n* = 51)	*p*-Value
Mean age (year)	66	67.6	0.138
L1–2	2 (3.3%)	4 (7.8%)	0.454
L2–3	14 (23.0%)	7 (13.7%)	0.138
L3–4	1 (1.6%)	12 (23.5%)	0.155
L4–5	13 (21.3%)	18 (35.3%)	1.000
L3–5	0 (0%)	7 (13.7%)	1.000
L2–5	0 (0%)	3 (5.9%)	1.000

Values are presented as mean ± SD and frequency (%), respectively. *p*-values correspond to independent *t*-test and Fisher’s exact test.

**Table 3:** Postoperative thigh symptoms in symptomatic group.

Thigh Symptoms	Symptomatic Patients	
	Post-Op Day 1	Post-Op 12 Months
Pain	20 (32.8%)	2 (3.3%)
Numbness	9 (14.8%)	0 (0%)
Weakness	18 (29.5%)	1 (1.6%)

**Table 4:** Thigh symptom group (*n* = 32).

Patient	Level	Symptoms		
		Pain	Numbness	Weakness
1	L1–2	+	+	+
Resolution (months)		No	12	3
2	L2–3	+	+	+
Resolution (months)		6	3	3
3	L2–3	+		+
Resolution (months)		3		1
4	L2–3			+
Resolution (months)				1
5	L2–3	+	+	
Resolution (months)		No	3	
6	L2–3			+
Resolution (months)				1
7	L2–3			+
Resolution (months)				1
8	L2–3	+		+
Resolution (months)		6		No
9	L2–3	+		+
Resolution (months)		3		1
10	L3–4		+	
Resolution (months)			3	
11	L2–3			+
Resolution (months)				1
12	L4–5	+	+	+
Resolution (months)		3	6	1
13	L2–3		+	
Resolution (months)			3	
14	L4–5	+		
Resolution (months)		3		
15	L4–5	+		+
Resolution (months)		12		6
16	L4–5			+
Resolution (months)				1
17	L3–4	+		+
Resolution (months)		3		1
18	L2–3			+
Resolution (months)				1
19	L4–5	+		
Resolution (months)		12		
20	L4–5	+		+
Resolution (months)		3		1
21	L2–3			+
Resolution (months)				1
22	L3–4	+		
Resolution (months)		3		
23	L4–5		+	+
Resolution (months)			6	1
24	L2–3	+		
Resolution (months)		3		
25	L2–3	+		
Resolution (months)		3		
26	L4–5	+		
Resolution (months)		6		
27	L2–3	+		
Resolution (months)		1		
28	L4–5		+	
Resolution (months)			6	
29	L4–5	+		
Resolution (months)		6		
30	L4–5	+		
Resolution (months)		3		
31	L4–5	+	+	
Resolution (months)		6	1	
32	L4–5			+
Resolution (months)				1

+ = presence of symptoms, No = permanent symptoms.

**Table 5:** Comparison of psoas major muscle volume between two patient groups.

	*n*	Psoas Major Muscle Volume (cm^3^)		*p* Value
		Mean ± SD	Median (Min, Max)	
**Pain**				
No	62	411.48 ± 113.95	420.30 (364.22, 531.31)	0.895
Yes	19	419.04 ± 84.32.	407.64 (365.93, 549.05)	
**Numbness**				
No	72	421.76 ± 108.80	420.30 (324.72, 514.16)	0.469
Yes	9	397.96 ± 118.41	407.64 (387.13, 552.46)	
**Weakness**				
No	63	411.40 ± 115.87	424.87 (324.72, 514.16)	0.887
Yes	18	419.67 ± 96.72	389.31 (351.30, 549.05)	

## Data Availability

Data Availability Statements in section “MDPI Research Data Policies” at https://www.mdpi.com/ethics (accessed on 10 March 2021).
